# Honor to Whom Honor Is Due

**Published:** 1881-01

**Authors:** 


### HONOR TO WHOM HONOR IS DUE.

THE complete exposure of the airy nothingness of the inert pillules known as high potencies is very generally credited to Milwaukee — to Milwaukee generously aided by sundry "Munchausen microscopists;" but we are happy to be able to announce that Philadelphia has a prior claim to this honor. For one of her physicians performed this Herculean task—cleansed this Augean stable, so to speak — not with his little spade, but with his little carbolic acid. Honor to whom honor is due! say we. This is a noble, a generous sentiment, yet we selfish mortals seldom willingly yield the honor due to genius.

Thus we remember how Harvey's discovery has been disputed, also Columbus', so too Hahnemann's; it has even been denied that Julius Cæsar ever exclaimed *veni, vidi, vici;* and so in this case, of equal historic importance, honor will, we fear, be bestowed very unwillingly on this modern Cæsar, who carbolic acid in hand, also exclaimed *veni, vidi, vici!* We can not give the exact date of this remarkable experiment and discovery (one only equalled in Philadelphia's history by Franklin's kite-flying), but we are fully justified in asserting its priority to the Milwaukee affair, which was indeed but a base imitation and as inferior to the original as a "paste" is to the "brilliant." The inner history of this momentous affair is now for the first time made known and let us hope the public will, for once, generously and promptly yield honor to whom honor is due! First let me state that this discovery was no "apple falling accident;" nay, it was solely due to the reasoning of a gigantic (eared) genius. Farmers foretell the "dropping" of a colt by the restless manner of the mare; so in this case, had any one been watching he would have seen how "coming events cast their shadow before," by the peculiar conduct of this—donkey. His sleep was bad, being prevented by an aggregation of ideas (too much *coffea?*) which were always repeated (*calc.*), but finally they settled into the one fixed idea (*graph.*), that he was a genius, a reformer, that his intellect was so immense as to make him feel quite as if he were double-headed (propably due to his dandified habit of using *musk*); this, we believe, was a decided mistake for the headless-body illusion (of *nux*) is much nearer the truth. Awakening from this restless sleep he longed for the company of his friends (Plumb) that he might give vent to his imaginations of fancy (Bell.); so he called together his friends, from the east and the west, from the north and the south, and then he opened his mouth and—brayed.

We pause here in this interesting narrative to offer another proof—if another be necessary—that this experiment differed from the Milwaukee fiasco; there they presented prominently the (*Phos.ac.*) symptom of seeing ciphers before the eyes, while the Philadelphia experimenter had pre-eminenfly the (*Bell.*) symtom of seeing insects in fluids. Being in a distructive mood, (*Merc. iod. Flav.*), he desired to destroy these insects, to drown them (*rhus.*) with his little carbolic acid. This was the experiment! Simple, neat, conclusive. Thus was it done; animalcules, a size smaller than whales—for he is no mean microscopist—were placed in tubs, and into these tubs solutions of carbolic acid, varying in strength, were cautiously injected by a Babcock's fire extinguisher. The strong solutions quickly destroyed these animalcules, but as he used more and more dilute solutions, this killing became slower and slower, and finally ceased. As soon as the vermin destroying point was reached this logical therapeutist announced to his admiring friends that there also ceased the health restoring power of drugs. Or in other words, no drug can cure disease when administered in a solution too attenuated to kill vermin. Eureka!! This test was called by its modest author—vermin-analysis! E. J. L.

